# Advanced Driver Assistance System Based on IoT V2V and V2I for Vision Enabled Lane Changing with Futuristic Drivability

**DOI:** 10.3390/s23073423

**Published:** 2023-03-24

**Authors:** K. Suganthi, M. Arun Kumar, N. Harish, S. HariKrishnan, G. Rajesh, S. Sofana Reka

**Affiliations:** 1 School of Electronics Engineering, Vellore Institute of Technology, Chennai 600127, India; 2 Department of Information Technology, MIT Campus, Anna University, Chennai 600025, India; 3 Centre for Smart Grid Technologies, School of Electronics Engineering, Vellore Institute of Technology, Chennai 600127, India

**Keywords:** advanced driver assistance system design, internet of things (IoT), data analytics, over-the-air, vehicle communication, machine learning

## Abstract

In conventional modern vehicles, the Internet of Things-based automotive embedded systems are used to collect various data from real-time sensors and store it in the cloud platform to perform visualization and analytics. The proposed work is to implement computer vision-aided vehicle intercommunication V2V (vehicle-to-vehicle) implemented using the Internet of Things for an autonomous vehicle. Computer vision-based driver assistance supports the vehicle to perform efficiently in critical transitions such as lane change or collision avoidance during the autonomous driving mode. In addition to this, the main work emphasizes observing multiple parameters of the In-Vehicle system such as speed, distance covered, idle time, and fuel economy by the electronic control unit are evaluated in this process. Electronic control unit through brake control module, powertrain control module, transmission control module, suspension control module, and battery management system helps to predict the nature of drive-in different terrains and also can suggest effective custom driving modes for advanced driver assistance systems. These features are implemented with the help of the vehicle-to-infrastructure protocol, which collects data through gateway nodes that can be visualized in the IoT data frame. The proposed work involves the process of analyzing and visualizing the driver-influencing factors of a modern vehicle that is in connection with the IoT cloud platform. The custom drive mode suggestion and improvisation had been completed with help of computational analytics that leads to the deployment of an over-the-air update to the vehicle embedded system upgradation for betterment in drivability. These operations are progressed through a cloud server which is the prime factor proposed in this work.

## 1. Introduction

Road safety has become a paramount concern issue in the present-day situation. The increase in the population of automobiles, the incidences of road accidents which increases day by day, and also the comfort in driving is a considerable factor along with these active safety features. ADAS (advanced driver assistance system) is a prominent feature in any modern automobile which enhances the vehicle to operate in a self-reliant mode without expecting many drivers or human intervention with improved safety levels [1,2]. Presently, ADAS are solely implemented for cruise control in most cases along with a few more utilities such as collision avoidance, navigation, lane changing detection, and a few other warning and indication mechanisms [2]. Yet, all these subsystems or features may not be integrated as a single entity in current ADAS and also the reliability or self-reliant operation of these systems does not meet the expectations. Even though several upgrades and improvisations were introduced in this system there are still plenty of enhancements that can be offered which will increase the overall safety and performance [3]. This work will be discussing a few of such enhancements with the integration of multiple technologies with the proposed ADAS design.

In the aspect of safety, this work uses computer vision-based lane change detection and proximity alert mechanisms to indicate the change in lane and to avoid collision [3,4]. There are already a few implementations available in both mechanisms but using computer vision yields more precision and sensitive alert indications. In this proposed work, the advancement of ADAS technologies is the connected car in which the vehicle intercommunication vehicle-to-vehicle (V2V), and cloud connection with vehicle-to-infrastructure (V2I) are completed [5]. Additionally, a drivability mode improvisation is proposed where we will continuously monitor critical parameters influencing the drive modes of an automotive, and based on this monitoring, the level of smooth drive can be indicated to the ADAS [6,7]. In addition, several custom modes of driving can be formulated for individual vehicles based on their nature of drive which is observed from the monitored parameters. The novelty of this custom driving mode formulation is that we could design the mode such that the ADAS can operate accordingly for different terrain regions and different traffic, and road conditions in an efficient way by adjusting the cruise, throttle, and operating factors as formulated [8]. By fusing this data with the vehicle’s sensor inputs it will become possible to create much more details of the surrounding area with real-time AI-based lane detection which can provide more accurate alerts and corrective actions, to avoid collisions [9].

In the proposed work, the collected information is stored in a local server and also in a cloud server. Grafana is used for the analytics and visualization of information with the help of this leads to future prediction. To achieve this, we use technologies such as computer vision, communication mechanisms such as V2V and V2I with the Internet of Things, and cloud analytics features with our ADAS [10,11]. Here, the role of computer vision is for providing lane change detection and collision avoidance as discussed before, whereas the V2V (vehicle-to-vehicle) technology is aided by this vision-based system to communicate the lane change and proximity alert between vehicles within a stipulated range [12]. Usage of this V2V communication improves the speed of communication with a high proneness to interference and other constraints. V2I (vehicle-to-infrastructure) technology is used to transmit the vehicular data and the data communicated in V2V to a hub which is a server in our application where the analytics on these data are performed [13]. Through V2I, individual vehicle data could be stored in a unique channel which is used for future analytics and driving mode formulation. The analytics mentioned are completed using a few cloud platforms where we could visualize and analyze the vehicle data collected which supports the formulation of the custom driving mode and also the level of smoothness in drive [14]. This data analytics and formulation along with vision-enabled safety system design is the proposed idea of the work which yields a better safety and reliant ADAS for modern automotive.

The ADAS can be enhanced more by working on futuristic works such as over-the-air updates (OTA). The cloud-connected vehicles are enabled to receive the system by upgrading to the ECU wherein the e software or firmware [15] updates are made based on the suggestion for custom mode suggestion for processing the data collected in various terrains through the OTA. These updates to the system with help of a cellular network by various analytics on cloud platforms are completed in the process [16]. At present, for ADAS the OTA or any other update is provided only for infotainment purposes whereas the update which is formulating serves the aspect of drivability improvement. The e OTA update transfer could also be categorized under V2I communication since the update is generated and transferred from common infrastructure to individual vehicles. The formulated custom modes from cloud analytics are transmitted to vehicles utilized by our ADAS by eradicating the need for additional hardware [16,17]. Utilizing these kinds of software-centric systems offers efficient ADAS even for low-cost vehicles in addition to the sophisticated luxury class of the vehicles which improves safety and reaches the industry’s goal for zero vehicle-related accidents, fatalities, and obsolete comfort for drivers. The advanced driver assistance system (ADAS) in an automotive is designed to serve the purpose of cruise control only during initial times, which was then aided with cognitive computing [4] and sensor clusters used in the emergency brake systems [18], drive beam, blind spot monitoring, auto parking, lane departure warning and assist to support the driving through increased safety and guidance [19,20,21,22,23,24,25,26,27,28,29,30,31,32]. The modern systems utilize intercommunication between vehicles, where the pedestrian detection and collision avoidance feature data were shared among vehicles to improve the error-prone systems in critical situations using different architectures such as V2V and V2I integrated with Internet of Things (IoT) structure [3] taking into consideration the different level security during communication.

In each generation, several subsystems such as ADAS using VANET protocol-based pedestrian detection with V2X [7], V2V-based lane change warning system [6], V2V-based crash detection and warning using vehicle position, context detection [23,33], YOLO (You Only Look Once)-based lane detection system using CSI camera [34] were developed where each of these systems offered a prominent and essential critical feature for a vehicle drive. Recent research focuses on integrating these subsystems using secure intercommunication with real-time protocols such as DSRC (dedicated short-range communication) and future 5G technologies [26]. The modern ADAS systems are expected to satisfy both the communication security and ease of using protocols along with the extensive features offered by our ADAS as high as possible. D2D (device-to-device) communication enabled V2V or V2X communication offering various QoS (quality of service) requirements 3D graph-based matching and hypergraph coloring-based resource block allocation to optimize the power consumed. Additionally, resource management can be optimized by the use of edge computing in GPU-based federated edge data centers and edge federation sizing through the ANN predictive power model [28] with a trade-off on the increased complexity of the system.

As advancement in the network, consensus-based vehicle control algorithms [17] and CAN-based communication for navigation assistance, electronic payment, and traffic updates [29] were also proposed. While using such modern sensors and communication protocols, we might be prone to vulnerabilities such as Sybil attacks, blackhole attacks, wormhole attacks, grey hole attacks, DoS attacks, DDoS attacks, GPS spoofing, jamming, malware attacks in the cyber aspect and spoofing, jamming, acoustic quieting, relay attack in sensors and cameras [30]. To deal with the security issues of these systems, a few field operational tests are performed suggesting a VANET in multi-hop mesh mode or routing algorithms for real-time [15]. In next-generation vehicles, the DSRC used for primary application can be replaced with a heterogeneous network integrating multiple wireless technologies such as Wi-Fi, LTE, WiMAX, and BLE through which these models can broaden the range with reduced latency communication [31]. One of the recent and emerging features of modern ADAS is by offering software updates for ECUs. There are few multicore virtualization technologies to perform SITU verifications to control the autonomous vehicle through a software update [9], to deliver these updates to the vehicles there are several technologies available such as mobile communication networks integrated with OEMs with recent 5G features [21], adaptive AUTOSAR with IoT connectivity in ECUs, these updates offered for critical bug fixing has to maintain interconnected system resilient against security threats [2]. The FOTA (firmware over the air) updates might reduce the complexity and downtime cost which can be used in our autonomous vehicles [25]. The fast and secure update package distribution with end-to-end security using ciphertext policy attribute-based encryption is completed, which can reduce overheads with better network load and also update the retrieval time management which is presently considered as the promising features experimented in this aspect of work.

The main contribution of this work compared to the literature exhibits the need of using communication protocols for automotive vehicles. This process involves the need for vehicle updates which are based on driver performance. This exhibits the utilization of effective IoT protocols and cloud architecture. The future outcome such as OTA based on the performance promotes cloud analytics and app-based firmware update. The proposed work also focuses on collision avoidance during lane change in autonomous vehicles which utilize the V2V protocol for information exchange. This proposed work determines the vehicle’s performance based on its own drive anomaly and also the IoT-based implementation, which will promote effective debugging through the communication protocol. Thus, implementing such protocols with the critical parameters data exchange will help in the advanced driver assistance system (ADAS) that will ensure the better performance of the vehicle. 

## 2. Proposed Design

The experimental purpose and futuristic insight of the ADAS for an electric vehicle are completed based on the various user-defined physical inputs using software such as Simulink in MATLAB [22]. The proposed methodology is based on the various inputs in the cloud infrastructure from which the behavior of electric vehicles for different drive cycles [24] in series, parallel and hybrid configurations [14] are analyzed continuously. The need for such prototyping is to optimize the powertrain requirements for different modes such as route-based drive, online drive, and integrated drive with path planning [11] and for driving mode selection [13] with the aid of ML algorithms for an economic drive [12]. To achieve this, in this work the process is analyzed with several critical parameters such as vehicle dynamics, motor and battery selection, speed, range, capacity, and weight estimations [10], and the impacts of different speed regions with critical parameter considerations [5] over the vehicle performance.

**Figure 1:**
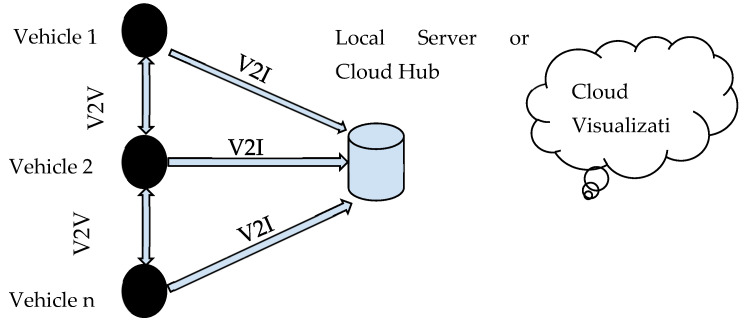
Overall system architecture.

The overall working system involves the end nodes, the control cloud hub, and the cloud database as mentioned in Figure 1. In the proposed work, the end nodes are the vehicles that are interfaced with the communication module. In this work, each vehicle sending its performance data such as state of charge (SOC), input power, average speed, and distance are calculated. For each set of vehicles, these data are sent. The hub or local server is placed in a junction where the process collects this data from the vehicle during an intersection. The local server is Influx DB which provides the feature of timestamp data management. These data are being utilized by the Grafana visualization data frame for visualization as well as analytics using machine learning.

### 2.1. Advanced Driver Assistance System 

**Figure 2:**
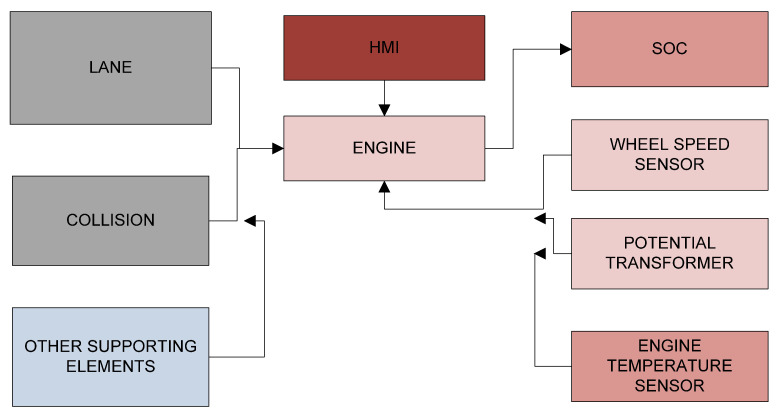
Block diagram of proposed advanced driver assistance system.

The proposed system exhibits the need for advancement to the present ADAS systems. This system, exhibited in Figure 2, establishes the critical parameters influencing the drivability of any automotive from which several custom driving modes with improved efficiency can be achieved at a greater level.

In any automotive, two major factors contributing to the performance of the vehicle are speed and fuel economy IC engine vehicles are considered these factors are given by wheel speed and fuel-to-air ratio or the energy used for commutating the engine. The proposed system in this work analyzes the performance of an electric vehicle by the way of monitoring the state of charge (SOC) of the battery, and input speed from the drive cycle of the electronic commutator. This is also completed with the power at either end of the motor-gear system connecting to the power controller to the vehicle wheel. At present, there are only two driving modes in automotive in the vehicle as sports and economy. In the sports mode the speed is given utmost importance which is suggested for long drives whereas in economy mode it is based on the fuel economic drive which is offered. The main work of this proposed system depicts to work these two modes of any automotive with several custom modes sophisticated to different scenarios based on the analysis results. From the perspective of safety, the proposed system is also aided by lane change detection which is another prominent feature of autonomous driving. A proximity alert can also be included in this aspect. After acquiring all the mentioned critical parameters through sensor clusters, the work uses e V2V and V2I communications to transmit the data between vehicles and also to the infrastructure where analytics is performed. Once the custom modes are designed from the OEM side, the model is incorporated with the process in the existing vehicle without any need for additional hardware through OTA (over-the-air) update [24,25] which would be the most promising feature of the proposed system and the unique analysis completed in the proposed model.

### 2.2. Vehicle-to-Vehicle and Vehicle-to-Infrastructure Communications

**Figure 3:**
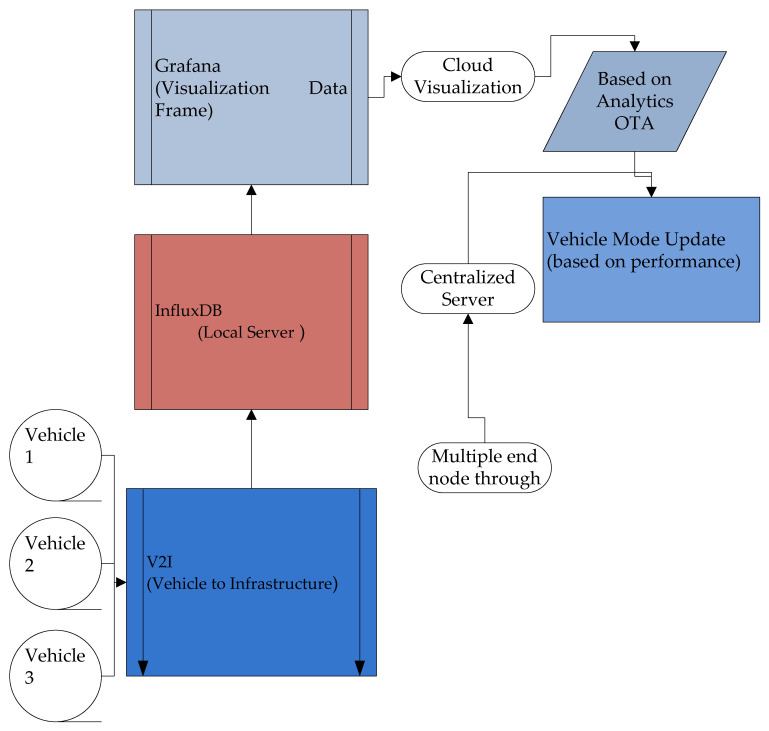
IoT functional flow diagram.

The communication between vehicles and also with infrastructure is implemented in real-time hardware. Generally, these communications are performed using a dedicated short-range communication protocol. In this proposed work, the process has been implemented in the system using node MCU which communicates using Wi-Fi (802.11) within a dedicated range and the data are transmitted between two vehicles which contributes to V2V and V2I communication. Vehicle-to-vehicle and vehicle-to-infrastructure) technology allows vehicles to communicate with other cars, infrastructure, and vulnerable road users to increase driver safety and smooth out the autonomous driving experience. In the proposed system implemented uses a LEVEL-6 IoT system where the individual vehicle will act as independent end nodes of sensing and actuating, the acquired data will be analyzed over the cloud or local server. The final result will be predicted after a cloud-based visualization. Figure 3 exhibits the IoT functional flow diagram stating the different parameters in the process after the V2V and V2I communication are made.

As proposed, the implemented system falls under the Level 6 IoT system where the vehicles act as end nodes communicating their dynamic data after sensing and actuating. Once the end nodes prepare their data using V2V and V2I communication concepts aided with Wi-Fi or Zigbee protocols the metadata are communicated to the local server of Influx DB from several hubs installed and programmed at given intervals. After acquiring all the vehicle data from hubs into a common ADAS database as shown in the output, it is subjected to visualization and analytics for prediction and alert operations. This is being taken care of by the Grafana Cloud visualization tool which gives us the state of the dynamic nature of vehicles to model future performance improvisations.

## 3. Results and Discussions

**Figure 4:**
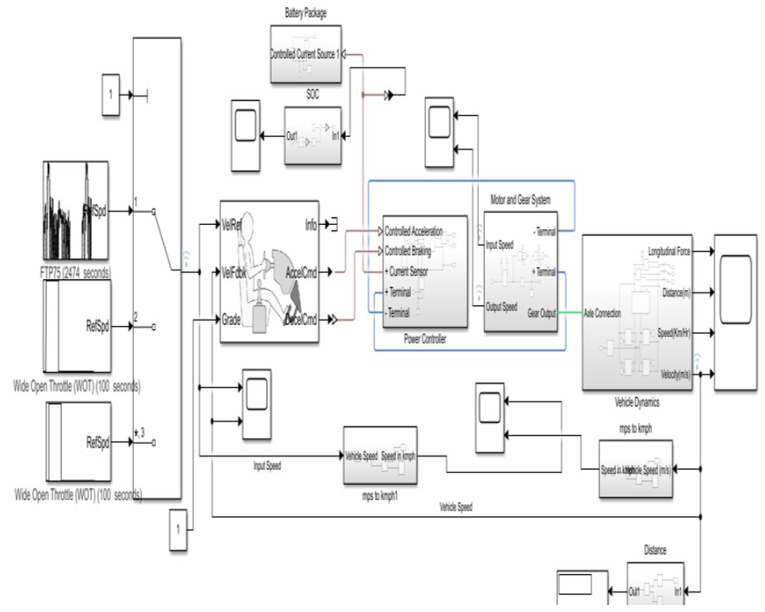
MATLAB Simulink EV design.

The entire work proposed has been completed with the aspect to the software and hardware codesign process for the system exhibited. To simulate a vehicle dynamic in a drivable model, the Simulink design in MATLAB. This simulation is implemented to generate real-time data during an electric vehicle drive cycle. These generated data are used as the performance metrics in our proposed work. Any vehicle parameters determine the vehicle performance such as SoC, traction rate, voltage input, and weight of the vehicle. The major four critical parameters of a car that has been monitored are input speed (speed from battery drive cycle), actual vehicle speed, SOC for the motor performance, and the power applied to motor gear from the power controller as the speed at the MG input system. Figure 4 illustrates in detail the drive cycle of an EV with desired input parameters given as a MATLAB Simulink EV design.

**Figure 5:**
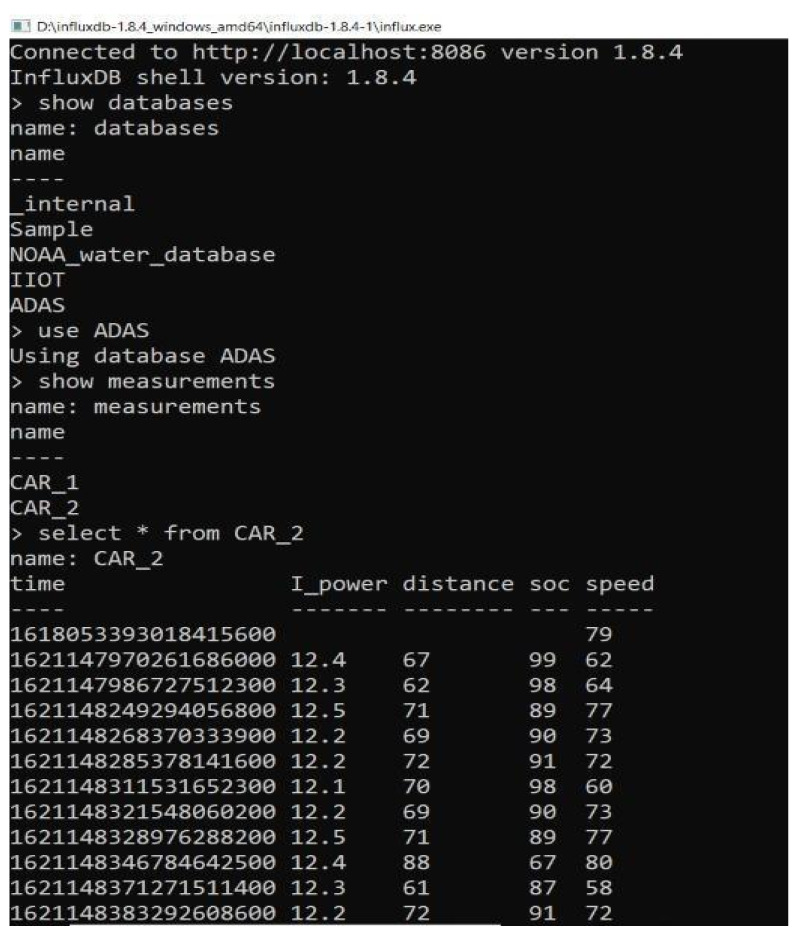
Collected data of CAR2 in the server.

For the proposed design, the Influx DB is the preferred local server that manages the sensor data collected through the V2I protocol. The console is a command-based query where the query is defined to create and manage the database. In similar ways, multiple tables are created for multiple end nodes in which the cars’ data are sent through the Influx DB. In this process, end node data are managed separately, through which the management of vehicle parameters is achieved. The Influx DB will be able to manage small to the massive amount of data that is required for cloud analytics and visualization in the design developed. Figure 5 represents the collected data of CAR 2 in the DB server for the process. The required data managed in the local server are exhibited as an image from the DB server.

**Figure 6:**
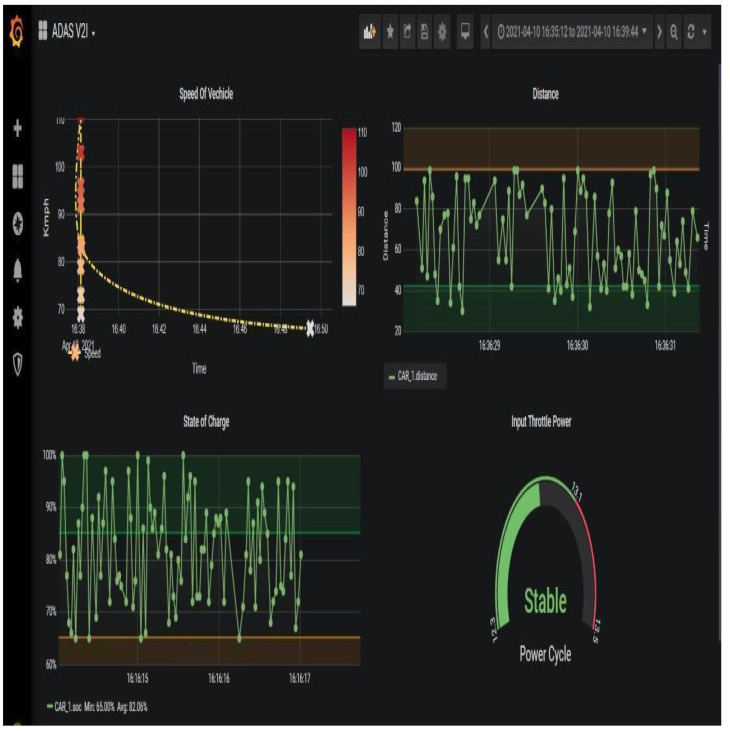
V2I data dashboard of CAR1.

In this process, to visually implement the data analytics for the process, Grafana is used to represent them effectively for the system. Grafana is a data frame that can be used to visually represent the data that are being recorded for the variation in the required time-stamped data for the entire process. This analysis as part of the proposed system shows a time series representation of the recorded data. In order to represent the process effectively Figure 6 the vehicle-to-infrastructure (V2I) data dashboard of CAR 1. It exhibits a clear understanding of the V2I dashboard representing the speed of the vehicle, distance, state of charge, and power cycle in the data visualization analysis.

**Figure 7:**
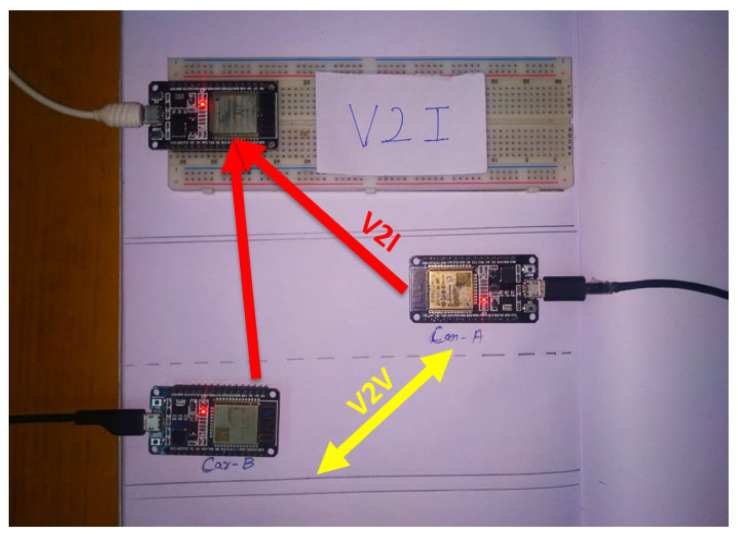
V2V and V2I hardware setup.

The hardware setup analysis is also completed with the computer vision analysis for the entire unit and is represented in Figure 7. In this hardware, the required analysis of V2I to V2V is completed accordingly. In this simulation, the ESP32 SoC controller is being considered as an electronic control unit (ECU) for other cars for wireless data sharing. For the gateway node (infrastructure) another ESP32 is used for the segregation of vehicle real-time data.

For the analysis to be performed with the hardware setup, real-time data sharing from CAR 1 to CAR2 and wireless data reception in CAR 2 are used. The wireless data reception on CAR 2 shows the V2V transmission between the two cars. It also shows the state of charge details, the range covered, and also the battery temperature related to the process communication. The tabulation screenshot figure shows the range between the two cars effectively with the process. The data reception to the local gateway node (ESP32) from both of the vehicles is also exhibited in the different ports at the gateway. Figure 7 exhibit the hardware setup for the communication process.

### Computer Vision Based Lane Detection and Lane Changing

The artificial intelligence (AI)-based lane detection model is simulated from the perspective of future scope where an autonomous vehicle detects a lane using the computer vision CMOS sensors (camera) based on deep learning and works on the centroid of the road in which an algorithm changes the lane position by an adaptive steering mechanism. The algorithm utilizes the OpenCV2 library using python language which is based on contour mapping, where the video is framed to detect the greyscale, based on which lane is being detected, and through which detection of lanes is performed.

- Turn left—centroid falls –ve degree
- Turn right—centroid falls +ve degree

**Figure 8:**
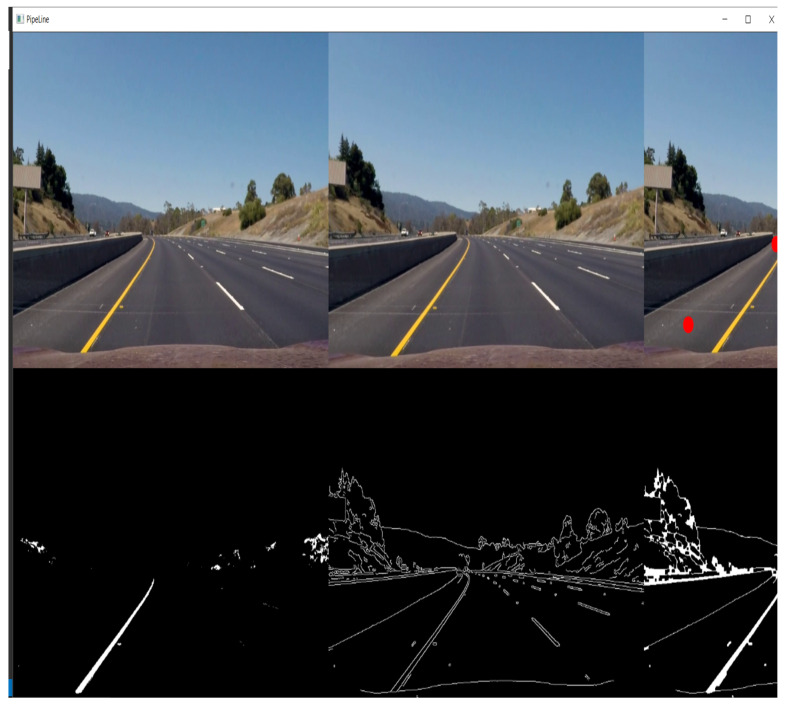
Pipeline stages involved in video processing for lane detection.

**Figure 9:**
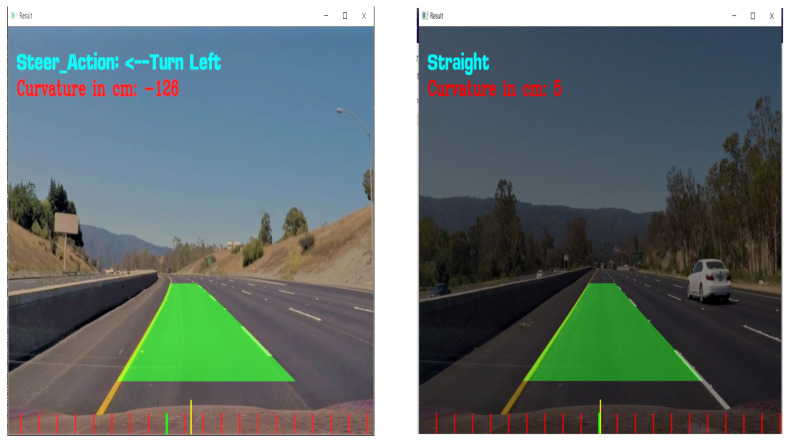

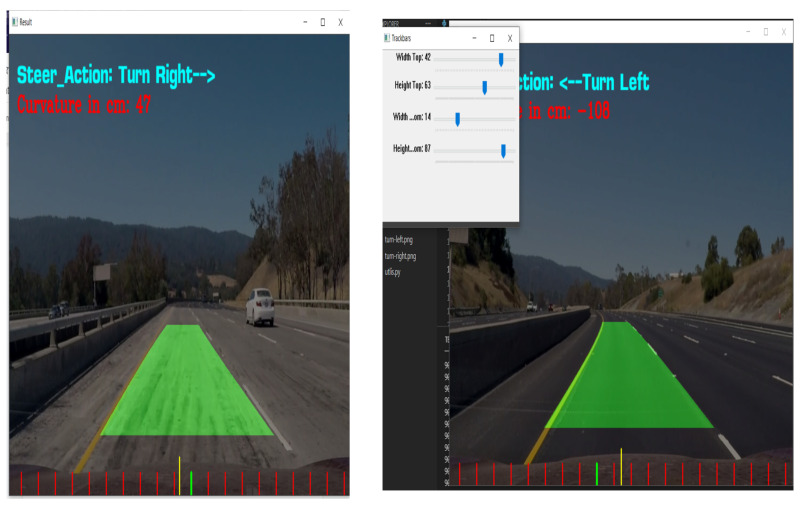
Lane detection frame from simulated video.

The core purpose of lane detection-based adaptive steering action for the implementation of V2V-based lane detection is implemented for effective lane changing and collision avoidance which ensures safe drivability. Figure 8 and Figure 9 represents the simulated figures of the model. The vehicle firmware update from the cloud app through the concept of OTA could be considered for performance in fewer vehicles. In the future, the system can be made reliable for both fuel-based as well as electric vehicles. In autonomous vehicles, V2I provides extra information to the vehicle’s existing navigation system. V2I uses a short-range wireless signal to communicate with compatible systems, and this signal is resistant to interference and inclement weather. In addition to safety benefits, V2I technology serves other purposes such as integrating automatic payments for tolls, parking, and similar fees.

## 4. Conclusions

The automotive system with futuristic functionality will pave the way for safety as well as comfort. The proposed work elevates the use of communication protocols such as V2V and V2I for futuristic automotive vehicles. By implementing such a system, critical vehicle updates are based on their drive performance. This is intended to utilize effective IoT protocols and cloud architecture for database management and cloud analytics. The future outcome such as OTA based on the performance promotes cloud analytics and app-based firmware update. The proposed work also focuses on collision avoidance during lane change in autonomous vehicles which utilize the V2V protocol for information exchange. We also determine the vehicle’s performance based on their own drive anomaly and also IoT-based implementation, which will promote effective debugging through the communication protocol. Thus, implementing such protocols with critical parameter data exchange will help in the advanced driver assistance system (ADAS) that will ensure the better performance of the vehicle.

## Data Availability

In this work, availability of data is not attached with the work.

## Nomenclature

ADAS	Advanced driver assistance system
V2V	Vehicle-to-vehicle
IoT	Internet of Things
ECU	Electronic control unit
BCM	Brake control module
PCM	Powertrain control module
TCM	Transmission control module
SCM	Suspension control module
BMS	Battery management system
V2I	Vehicle-to-infrastructure
OTA	Over-the-air
